# Forms of support giving and receiving, and their associations with self-rated health and general self-efficacy among older adults in Switzerland

**DOI:** 10.3389/fpsyg.2026.1759995

**Published:** 2026-03-03

**Authors:** Shkumbin Gashi, Heidi Kaspar, Martin grosse Holtforth

**Affiliations:** 1 Department of Psychotherapy and Clinical Psychology, Institute of Psychology, University of Bern, Bern, Switzerland; 2 School of Health Professions, Bern University of Applied Sciences, Bern, Switzerland; 3 Department of Neurology, Psychosomatic Medicine, Inselspital, Bern University Hospital, Bern, Switzerland

**Keywords:** functional ability, general self-efficacy, intrinsic capacities, older age, self-rated health, social environment, support

## Abstract

Our aim was to examine associations between determinants of functional ability as defined by the World Health Organization: intrinsic psychological domain captured by general self-efficacy and self-rated health, and social environment captured by support given and received. 286 Swiss-based older adults, recruited via multiple channels, completed a web-based survey. Older adults reported providing more support than they perceived receiving, particularly within informal networks of spouses/partners, children, friends, and neighbors. They also reported high levels of general self-efficacy and good to very good self-rated health. Out of thirteen forms of support giving and receiving, only providing support to friends/acquaintances and to fellow club/association members correlated positively with general self-efficacy. Self-rated health showed negative associations with receiving support from various sources, but was unrelated to providing support. On the basis of these results, we propose new avenues for examining associations between social support, general self-efficacy, and self-rated health.

## 1 Introduction

The global population is aging rapidly and consistently. According to the United Nations' (2019) demographic projections, the number of adults aged 60 years and older is expected to surpass 2 billion by 2050. This UN ongoing demographic shift appears to have wide-ranging implications with regard to institutional and policy responses across healthcare, labor markets, and social security systems. However, oftentimes approaches to old age reflect what the sociologist Townsend (1981) calls and criticizes as “structured dependency,” which frames older age in terms of decline, vulnerability, and burden leading to questions such as: *How will the rapid aging of the population strain healthcare and social security infrastructures? Who provides care at home, and at what personal and monetary cost?* Despite the relevance of these questions, this perspective on older age, often overlooks the capacities, heterogeneity, and agency of older adults. Contrary to this limited framing, the World Health Organization (WHO) has been advocating for a more holistic approach to older age through the concept of *functional ability*, particularly highlighted in the WHO’s (2020) campaign/framework *Decade of Healthy Aging 2021–2030*. Within this framework, the WHO (2020) defines functional ability as the combination and interaction of one’s five intrinsic capacity domains (locomotion, vitality, sensory, cognition, and psychological) with one’s environments (i.e., social, structural) that enable older adults to live well and be healthy. By introducing the concept of functional ability, the WHO moves beyond defining health solely through the presence or absence of disease, but focuses on the overall functioning of individuals across various life domains, from meeting the most basic needs to growing emotionally and intellectually, to being active members of society (WHO, 2020). Increasingly, evidence supports this approach and framework that recognizes older adults’ agency, showing that today’s older adults exhibit higher levels of functional independence, labor force participation, digital engagement, and physical activity, along with increased healthy life expectancy (Beltrán-Sánchez et al., 2015; Diehl et al., 2020; Vilhelmson et al., 2022). The WHO (2020) therefore calls for research on the concept of functional ability to further advance a holistic and emancipatory view of older age. We respond to this call by examining the associations between determinants of functional ability, intrinsic psychological domain (general self-efficacy, self-rated health) and social environment (support given and received). The three central concepts of our study are operationalized as follows: We have operationalized social support as the acts of providing and/or receiving support in everyday life, with sources ranging from informal personal ties, to civic and peer organizations, and professional/institutional services. Self-efficacy refers to an individual’s belief that they have the ability to persevere and complete specific tasks (Bandura, 1994), while self-rated health is defined as the overall perception an individual has of their own health (Bardage et al., 2005).

### 1.1 Receiving and providing support

With the expanding diversification of support systems, older adults find themselves both providing support to others, while also receiving various forms of support themselves. In Switzerland, older adults support the society substantially, for example, by investing approx. 157 million hours in 2024 in caregiving duties for their grandchildren (Swiss Federal Statistical Office, n.d.), and 54 million hours in voluntary activities in 2016 (Swiss Federal Statistical Office, 2024b). At the same time, older adults benefit greatly from diverse support systems, i.e., receiving assistance from family caregivers (Swiss Federal Office of Public Health, 2018), relying on costly healthcare services (Chastonay et al., 2018), and receiving additional “in home” services and forms of support, including community-based approaches. For example, the concept of “caring communities” has recently received considerable attention, ranging from an enthusiastic welcome to critical observation (i.e., Kaspar and Schürch, 2024; Schürch and van Holten, 2022).

International empirical evidence has highlighted the significant relevance of support for older adults’ wellbeing, with both receiving and providing support being positively associated with psychological resilience. Receiving support, particularly social and informal support has the potential to yield diverse benefits for older adults. Literature has documented its relation to diverse aspects of mental health, such as life satisfaction (Dong et al., 2024), loneliness (Chen and Feeley, 2013), depressive symptoms (Tsuboi et al., 2016), etc. Similarly, providing support to others, both to family members and to people beyond kin, is also related to better wellbeing. For instance, those older adults who engage in supporting others (i.e., volunteering) perceive themselves as more healthy (Abolfathi Momtaz et al., 2013), with volunteering being positively associated with life satisfaction (Gil-Lacruz et al., 2019), optimism and purpose in life, and lower levels of hopelessness and of depressive symptoms (Kim et al., 2020). Some studies have concluded that providing support is more strongly related to wellbeing than receiving it (i.e., Thomas, 2010; Zanjari et al., 2022). In this rich social context, where support is both received and provided by older adults, a range of systems and forms of support are utilized being both informal and formal. On one side, informal support flows between family, relatives, friends and neighbors, while on the other, healthcare professionals, social service organizations and government programs provide formal support. These diverse systems may interact in complementary, substitutive, or conflicting ways.

A significant body of evidence is focused on the association between formal and informal care (i.e., Armi et al., 2008) and interactions between its relevant actors, yielding heterogeneous results. While informal caregivers are considered a valuable supporting resource for medical professionals (Boros, 2010; Dal Bello-Haas et al., 2014), this partnership does not materialize often enough, potentially because many professionals consider themselves as being the experts (McWilliam et al., 2001). However, very often, informal caregivers take the roles of professional caregivers (Lyu et al., 2024). Other studies look at overlaps between less formal and more social types of support, reporting associations between providing care from grandchildren and receiving support from children (Jahangir et al., 2025), caregiving for grandchildren and voluntary community engagement (Bulanda and Jendrek, 2016), support that neighbors exchange with one another (Seifert and König, 2019), etc.

While the body of evidence has provided a better understanding of the relationships between informal and formal care, and between other forms of support, it has largely been dyadic in approach. However, care and support are rarely one-to-one but rather emerge from a dynamic relationship between multiple actors and systems. Often, older adults rely on a combination of family members, friends, neighbors, volunteer groups, professional carers, and institutional care, while at the same time giving support to others in various forms, be that through grandchildren care, volunteering, etc. Given that support in older age extends beyond traditional caregiving and one-to-one interactions, the literature would benefit from a broader view of how different forms of support are exchanged across multiple systems. Hence, in the present study, we analyze the bivariate association between seven forms of providing and six forms of receiving support, across various support systems (see Table 1 for all sources). We classify the support systems, which we characterize in our study by who provides the support and how organized they are: Informal personal ties, civic & peer organizations and professional/institutional services.

**Table 1:** Forms of providing and receiving support categorized by support systems.

Support system	Providing support (PS)	Receiving support (RS)
Informal personal ties	Spouse/partner; children; grandchildren; other relatives; friends/acquaintances; neighbors	People from family/acquaintances (friends, neighbors, etc.)
Civic & peer organizations	Fellow club/association members	Volunteers (neighborhood assistance, religious community, etc.); family caregiver groups *(peer support through support group, online forums, etc.)*
Professional & institutional services	–	Medical/health professionals (doctor, nurse, psychologist, etc.); social services professionals; counseling/contact/information center *(e.g., Pro Infirmis, municipality)*

### 1.2 General self-efficacy

Self-efficacy by Bandura (1994) refers to the belief that a person has in their ability to complete a particular task. Bandura (1994) argues that self-efficacy is specific to particular tasks and contexts and cannot be generalized across all situations. According to this logic, a person who scores high on a scale measuring mathematical efficacy may feel confident in completing mathematical tasks, but that score does not necessarily mean the person also feels confident in completing tasks in unrelated domains, such as physical or social tasks. This task-specific approach has significantly shaped and directed self-efficacy research to this day. For instance, researchers who aim to examine how confident a specific group of individuals are in their ability to complete physical exercises typically use a concrete physical exercise self-efficacy scale, in accordance with Bandura’s task-specific principle (i.e., McAuley et al., 2003; Miller et al., 2019; Warner et al., 2011, 2014, etc.). However, other researchers have suggested that self-efficacy can be considered not only in relation to specific tasks but also as a more general, trait-like belief in one’s ability to perform across a variety of situations. Hence, researchers such as Schwarzer and Jerusalem (1995) have developed the General Self-Efficacy Scale (GSE), which has cross-cultural universality, as shown by Scholz et al. (2002), and which, according to Luszczynska et al. (2005), shows significant correlations across individuals from different countries with indicators of psychological wellbeing such as optimism, self-regulation, self-esteem, etc. Research evidence on general self-efficacy (GSE) in older age in relation to providing support is mostly informed by and focused on health from a medical perspective. An example of such research is the meta-analysis of Whitehall et al. (2021), which identified 40 studies (*n* = 4,731) that compared the levels of general self-efficacy scores between those older adults who have received care services and those who did not as well as between individuals receiving care services from diverse sources and forms like community care, inpatient care, and outpatient care. Results showed that individuals receiving care are more likely to report a lower general self-efficacy than those who did not receive any form of care. Moreover, individuals who received acute medical inpatient care reported the lowest GSE, while those receiving care provided by primary care providers had the highest GSE scores. As noted earlier, evidence on associations between general self-efficacy and community life of older adults, be it social participation or support exchanges has been shaped by the debate over whether self-efficacy is task-specific or general. As a result, most studies tend to use specific measures assessing social participation (i.e., Oe and Tadaka, 2023), support-self-efficacy scales (i.e., Wu and Sheng, 2019), or other specific scales, such as self-management and initiative-taking self-efficacy scale (Alma et al., 2012), etc.

Few studies have taken the opposite approach by focusing on general self-efficacy, as described below. Hosseingholizadeh et al. (2019) conducted a study with 456 older members of community centers in Iran and found that GSE and received support (captured by the size of one’s support network and satisfaction with that network), both associated with increased social participation (i.e., visiting neighbors, joining associations). Okoye et al. (2022) studied a sample of 100 community-dwelling older adults and found significant correlations between general self-efficacy and support received initially, however these associations became non-significant when controlling for demographic variables such as age, sex and education. Last, Kwon et al. (2024) found that during the COVID-19 pandemic, general self-efficacy mediated the associations between received social support and feelings of hopefulness in 222 adults 50 years and older. Taking into account that most studies focus on task-specific self-efficacy, tend to examine support from a medical or care-setting perspective, or rely on narrow indicators of support, we argue that there is still a gap in evidence on how general self-efficacy relates to the broader ecology of support exchanges in older age. In particular, we lack substantial evidence on whether general self-efficacy is associated across multiple support sources, or only with specific forms, and whether this applies similarly to both giving and receiving support across settings (informal personal ties, civic & peer organizations and professional/institutional services). To address this gap, we explore correlations between general self-efficacy and 13 forms of support, both provided and received, across various sources (see Table 1).

### 1.3 Self-rated health

According to the Swiss Federal Statistical Office (2025a), around 79% of Swiss residing adults aged 65–74 years report very good health, while this figure decline to 69% among those 75 years and older.

These rates of self-rated health may be explained by and attributed to socio-economic, educational, and lifestyle factors, all of which contribute to creating a favorable living context. Most Swiss-residing older adults report that they are satisfied with their financial situation (Swiss Federal Statistical Office, 2025b), exhibit high levels of mobility, frequently participate in outdoor activities (Fischer et al., 2021a, 2021b), are actively involved in voluntary work, contribute to community life, and maintain social connections (Lamprecht et al., 2020). Switzerland seems to perform well in other key indicators of aging as well. Life expectancy at birth is among the highest globally (Swiss Federal Statistical Office, 2024a), and Swiss-residing older adults perceive old age to begin at around 80 years (Swiss Federal Statistical Office, 2024b), which stands in contrast to the widely recognized gerontological benchmarks of 60–65 years. Research has consistently highlighted the importance of social support in influencing self-rated health (SRH) among older adults, with a predominant focus on the effects of receiving support. What connects the existing evidence is the idea that even more important than the presence of support, is how that support is experienced, perceived, and reciprocated.

Available literature shows that support is both objective, measured by its quantity and quality, and subjective, defined by how adequate it is perceived by those who receive it. For instance, a much earlier study by Krause (1987) with U.S. Texas-based older adults showed that, irrespective of the amount of support they received, those who found that the support they received was not matching what they thought they needed in that moment reported worse self-rated health. This was in comparison to those who were satisfied with the adequacy of the support they were receiving.

White et al. (2009) results also highlighted the perceived adequacy of emotional support received to be particularly relevant. Those who felt that the amount of support they received was not enough, reported worse self-rated health compared to those who perceived the support available to them as being enough. The idea of support being closely linked to how it is perceived is also explained by the model of thriving through relationships by Feeney and Collins (2015). Among other aspects, this model explains how receiving support is much more than simply satisfying a care and support-based need. What they call “insensitive or unresponsive support” can actually be threatening for one’s psychological functioning. Another aspect explored in the literature is how diverse forms of support are associated with better self-rated health, with receiving emotional support showing consistent positive associations with self-rated health (i.e., White et al., 2009; Bélanger et al., 2016; Zunzunegui et al., 2001), while evidence in other forms is less conclusive. For example, some studies have found receiving instrumental support to be positively associated with self-rated health (i.e., Mao et al., 2020; Zunzunegui et al., 2001), others have found negative associations between the two, particularly among men (Li et al., 2009). While emotional support may not necessarily be related to dependency, instrumental support, as argued by Mao et al. (2020), is related to having more complex needs connected to care.

Research that has studied diverse systems of support and compared them, particularly the differences between kin and non-kin networks, has shown that in general, more diverse social networks are associated with higher self-rated health (Piedra and Iveniuk, 2025). Moreover, while both kin and non-kin related support are associated with better self-reported health, the non-kin one has shown stronger associations (i.e., Merz and Huxhold, 2010; Chopik, 2017). When support is reciprocal, older adults tend to show stronger associations with self-rated health when they perceive that the support they provide is more than what they receive. This applies both in relation to family members (Liu et al., 2024) and when extended to non-kin interactions in the community (Abolfathi Momtaz et al., 2013). Despite the existing evidence on how support is perceived, and on differences between emotional and instrumental support, kin and non-kin, and giving versus receiving support, the literature is still inconclusive and has gaps. And we have identified particularly two: (a) existing literature is mostly focused on receiving support, instead of accounting for reciprocity (giving and receiving); and (b) comparisons of support based on informal personal ties, civic and peer organizations and professional/institutional services within the same model remain largely unexplored. Hence, we aimed to contribute to covering this gap by analyzing pairwise (zero-order) associations between self-rated health and 13 forms of support as listed in Table 1.

### 1.4 Present study

Our overall aim in this study was to examine associations between two determinants of functional ability (WHO, 2020); the intrinsic psychological domain captured by general self-efficacy and self-rated health and the social environment captured by support given and received.

To make these associations interpretable, we first aimed to understand the overall level and distribution of these two determinants in our sample.

Hence, research question 1 to 4 address the first and second aim (description of determinants), whereas the 2 hypotheses address the third and main aim of the study (associations between the determinants of functional ability).

Our specific aims were:

Aim 1: Social environment as a determinant of functional ability. Our first aim was to examine the frequency and patterns of support exchange among older adults across informal personal ties, civic and peer organizations, and professional or institutional services, both given and received. For this aim we have compiled two research questions:

*RQ1*. How frequently do older adults provide and receive support across diverse sources?

*RQ2*. What are the correlations among different forms of giving and receiving support across sources?

Aim 2. Intrinsic psychological domain as a determinant of functional ability. Our second aim was to assess the intrinsic psychological domain among older adults, represented in our study by general self-efficacy and self-rated health. For this aim, we have compiled the research questions:

*RQ3*. What are the levels of general self-efficacy among older adults?

*RQ4*. How do older adults rate their own health?

Aim 3. Associations between determinants of functional ability. Our third and main aim was to examine how the social environment and the intrinsic psychological domain are associated with one another as determinants of functional ability. For this aim, we have tested two hypotheses:

*H1*. Higher levels of receiving and providing support are associated with higher self-rated health among older adults.

*H2*. Higher levels of receiving and providing support are associated with higher general self-efficacy among older adults.

## 2 Methodology

### 2.1 Study design and procedure

This is the third and final study in a series investigating the WHO’s (2020) concept of functional ability. In the 1st study, we have used a qualitative approach to explore how participatory action research (PAR) can enable older adults to expand functional ability through enriched relationships, intellectual engagement, and meaningful social roles (Gashi et al., 2023). In the 2nd study (Gashi et al., 2025), we studied the associations between social participation and the satisfaction of basic psychological needs, while in the current study, we shifted our focus toward relationships between other constructs: support receiving and giving, general self-efficacy, and self-perceived health.^1^ We employed a cross-sectional design, gathering the data using a self-administered web-based survey through ILIAS.^2^ Participants had to be 65 years or older, reside in Switzerland, and speak German.

The final sample consisted of 286 older adults who met inclusion criteria. We have structured and organized the recruitment process around a purposive, multi-platform strategy to maximize the probability of reaching diverse individuals, including formal educational institutions, national portals for older adults and broad social media platforms.

We made the survey user-friendly by making it accessible on desktops, tablets, and mobile devices. Before distributing the survey, we conducted a small-scale pilot test (*n* = 6) with individuals from the target population to test and improve the item clarity and compatibility with the Swiss context. Once the participants clicked the link to the survey, they were presented with an introductory information section, which outlined the study aims, voluntary nature, anonymity protocols and data handling procedures. Participation was entirely voluntary, and participants could skip any question without justification or obstacles to proceed to the next item. They were informed that no personally identifiable information would be collected and that the submitted data could not be withdrawn post-submission due to anonymization. We exported the survey data responses from ILIAS in comma-separated values (CSV) format, allowing for high fidelity data extraction and maintaining compatibility with statistical software used to analyze data in our study, which in our case was IBM SPSS v. 27 (IBM Corporation, Armonk, NY, United States).

### 2.2 Participant demographic profile

In our study, 46.9% of participants had an age between 65 and 70 years, while those over 81 years old were less represented, at only 4.9%. The gender distribution showed a dominance of women (64%) over men (36%), while regarding the education 34.3% held vocational or technical qualifications and 31% completed university-level education. Regarding their retirement status, 66.1% reported being fully retired, while 79.4% described their financial situation as good or very good. Taking into account all these demographical characteristics, our sample can be described as a sub-population which is compiled of community-dwelling older adults, predominantly aged 65–75, with moderate to high educational attainment and relatively stable financial conditions (Table 2).

**Table 2:** Sociodemographic characteristics of participants (*N* = 286)^a^.

Variable	Response category	*n*	%
Gender	Female	183	64.0
	Male	103	36.0
	Other	0	0
Age	65–70	134	46.9
	71–75	85	29.7
	76–80	53	18.5
	Over 80	14	4.9
Citizenship	Swiss	252	88.1
	Dual (Swiss and other)	22	7.7
	Non-Swiss	10	3.5
	Missing	2	0.7
Civil status	Married	145	50.7
	Cohabiting	26	9.1
	Living separately	6	2.1
	Single	22	7.7
	Divorced	47	16.4
	Widowed	34	11.9
	No answer	6	2.1
Household	Living alone	115	40.2
	With spouse/partner	163	57.0
	With child(ren)	2	0.7
	With other relatives	2	0.7
	With other people	3	1.0
	No answer	1	0.3
Education	Mandatory schooling	7	2.4
	Vocational training	98	34.3
	Secondary school	28	9.8
	Higher technical school	62	21.7
	University	91	31.8
	No answer	0	0
Financial situation	Manage very well	107	37.4
	Manage well	120	42.0
	Just manage	32	11.2
	Restrict myself	20	7.0
	Restrict a lot	5	1.7
	No answer	2	0.7
Retirement status	Fully retired	5	1.7
	Self-employed	14	4.9
	Part time employed	74	25.9
	Not employed	189	66.1
	No answer	4	1.4

a The same table is presented in the 2nd study, Gashi et al. (2025), for reasons elaborated above in the procedure (see text footnote 1).

### 2.3 Survey instrument

We organized and structured the survey into four sections: (a) demographic background, (b) social support, (c) self-rated health status, and (d) general self-efficacy. We adapted the items from Grillenberger (2016) to address the demographic background of our participants through variables such as age, gender, marital status, living arrangements, household composition, etc. Self-rated health was captured through a single item, as practiced in other studies (i.e., Caramenti and Castiglioni, 2022), rated with a five-point Likert scale response format, ranging from poor to excellent. We adapted items from Kaspar et al. (2019) to assess received social support with items including support from family/acquaintances, volunteers, medical/health professionals, social services professionals, counseling/contact/information centers, and other caregiving relatives. We also added items on providing support, such as to spouse/partner, children, grandchildren, other relatives, friends/acquaintances, fellow club/association members, and neighbors. We recorded responses based on a five-point Likert scale (1 = never, 5 = daily). Last, we measured general self-efficacy through the 10-item General Self-Efficacy Scale by Schwarzer and Jerusalem (1995). This scale has demonstrated strong psychometric properties across international samples, with Cronbach’s alpha ranging from 0.76 to 0.90 (Schwarzer and Jerusalem, 1995). In our study, the reliability was *α* = 0.90.

### 2.4 Analysis

To assess the reliability of the General Self-Efficacy Scale, we used Cronbach’s α. As the first step to understand and present general trends in our data, we used descriptive statistics, including frequencies, distributions, means (M), standard deviations (SD), minimum (Min), and maximum (Max) values. We performed Spearman’s rank correlations to examine pairwise associations between receiving and providing support forms and a factor analysis to identify the latent factors underlying the diverse forms or received and provided support. We also conducted the Spearman’s rank correlations to analyze associations between support and variables such as self-rated health and general self-efficacy, a Kruskal-Wallis test to examine differences in support categories across self-rated health levels and a Jonckheere-Terpstra to assess trends in general self-efficacy based on specific forms of support.

## 3 Results

### 3.1 Trends in support giving and receiving

Older adults in our study are more often providers of support than recipients. Forty (40) of them reported that they provide support to their spouse/partner at least several times per week, and 99 do so on a daily basis. Sixty-one (61) provide support to their children, 53 to grandchildren, 60 to friends/acquaintances, and 45 to neighbors, at least once a month. In contrast to providing, the frequency of receiving support is lower, particularly from civic/peer and formal sources of support. For example, 198 participants reported never receiving support from a counseling/contact/information center, 167 never received support from volunteers, 212 never received support from family caregiver groups and 211 from social service professionals. People from family/acquaintances are the most consistence source of support, but even here, to a smaller extend, with only 26 reporting to receive support monthly and 17 several times per week. In most forms of receiving support, “never” is the most common response, suggesting that many older adults receive minimal assistance across all available resources. In contrast, “never” is much less frequent in forms of providing support (see Table 3 for details).

**Table 3:** Frequency of providing and receiving support among older adults across different source.

Support source	Never	Rarely	Once a month	Several times per week	Daily	Valid
						*N*
Spouse/partner (PS)	54	27	12	40	99	232
Children (PS)	56	97	61	33	5	252
Grandchildren (PS)	90	60	53	24	4	231
Other relatives (PS)	87	124	32	10	0	253
Friends/acquaintances (PS)	27	167	60	10	2	266
Neighbors (PS)	42	163	45	11	2	263
Fellow club/association members (PS)	102	103	29	7	0	241
People from family/acquaintances (friends, neighbors, etc.) (RS)	66	121	26	17	20	250
Volunteers (neighborhood assistance, religious community, etc.) (RS)	167	51	9	5	1	233
Medical/Health professionals (doctor, nurse, psychologist, etc.) (RS)	100	126	11	3	2	242
Social services professionals (RS)	211	14	3	1	2	231
Family caregiver groups *(peer support through support group, online forums, etc.)* (RS)	212	18	3	1	4	238
Counseling/contact/information center *(i.e., Pro Infirmis, municipality)* (RS)	198	33	1	1	2	234

PS is short for providing support; RS is short for receiving support. Valid *N* is reported for each item because some support sources do not apply to all participants (i.e., someone may not have grandchildren).

### 3.2 Descriptive statistics of general self-efficacy

The general self-efficacy scores among the participants showed a mean (M) of 31.92 and a standard deviation (SD) of 4.52 (Table 4).

**Table 4:** Descriptive statistics for general self-efficacy scores among older adults.

Variable	*N*	Minimum	Maximum	Mean	Std. deviation
General self-efficacy	273	10.00	40.00	31.9231	4.51687

Out of 286 individuals surveyed, 273 participants were included in the analysis as they provided complete responses for the general self-efficacy measure.

### 3.3 Distribution of self-rated health

The majority of participants in our study reported positive self-rated health, with 45.8% rating their health as good, 34.3% as very good, and 9.1% as excellent. Negative perceptions of self-rated health were minor, with only 9.4% reporting not so good health and 1.4% reporting bad health (Table 5).

**Table 5:** Frequencies of Self-rated health status among older adults.

Variable	Bad		Not so good		Good		Very good		Excellent	
	*N*	%	*N*	%	*N*	%	*N*	%	*N*	%
Self-rated health	4	1.4	27	9.4	131	45.8	98	34.3	26	9.1

### 3.4 Interconnections between receiving and providing support: a correlation analysis

We have observed multiple correlations between 13 forms of providing and receiving support included in our study. Based on Cohen’s (1988) effect size criteria (small r = 0.10, medium r = 0.30, and large r = 0.50), these correlations fell across three effect size levels. The highest correlations were noticed between supporting children and grandchildren (0.646), supporting neighbors and supporting friends/acquaintances (0.510), while medium correlations were noticed between receiving support from family/acquaintances (friends, neighbors, etc.) and from health/medical professionals (0.455), receiving support from counseling/contact/information center with receiving support from family caregiver groups *(peer support through support group, online forums, etc.)* (0.443) and from social services professionals (0.386). Providing support to fellow club/association members correlated with supporting friends/acquaintances (0.410), and with supporting neighbors (0.347) and receiving support from volunteers correlated positively with supporting neighbors (0.366). Other correlations (Table 6), including those who fall on the small effect sizes, are listed in Table 6, pointing to the process of receiving and providing support being highly complex and interconnected.

**Table 6:** Correlations between receiving and giving support.

Variable	1	2	3	4	5	6	7	8	9	10	11	12	13
1	–	0.347**	0.306**	0.366**	0.140*	0.144*			0.186**	0.189**		0.254**	0.510**
2	0.347**	–	0.180**	0.198**		0.162*	0.203**	0.137*	0.248**			0.183**	0.410**
3	0.306**	0.180**	–	0.400**	0.455**		0.225**	0.264**	0.237**	0.177**		0.200**	0.261**
4	0.366**	0.198**	0.400**	–	0.358**	0.312**	0.336**	0.270**	0.196**			0.227**	0.313**
5	0.140*		0.455**	0.358**	–	0.362**	0.352**	0.288**	0.150*	0.150*		0.160*	
6	0.144*	0.162*		0.312**	0.362**	–	0.412**	0.386**		0.174*			
7		0.203**	0.225**	0.336**	0.352**	0.412**	–	0.443**					
8		0.137*	0.264**	0.270**	0.288**	0.386**	0.443**	–					
9	0.186**	0.248**	0.237**	0.196**	0.150*				–	0.282**	0.246**	0.210**	0.182**
10	0.189**		0.177**		0.150*	0.174*			0.282**	–	0.646**	0.212**	
11	.								0.246**	0.646**	–	0.176**	
12	0.254**	0.183**	0.200**	0.227**	0.160*				0.210**	0.212**	0.176**	–	0.243**
13	0.510**	0.410**	0.261**	0.313**					0.182**			0.243**	–

*p* < 0.05 (*), *p* < 0.01 (**).

1 = Support to neighbors; 2 = support to fellow club/association members; 3 = Receiving support from family/acquaintances (friends, neighbors, etc.); 4 = Receiving support from volunteers; 5 = Receiving support from health/medical professionals; 6 = Receiving support from social services professionals; 7 = Receiving support from counseling/contact/information center; 8 = Receiving support from family caregiver groups (peer support through support group, online forums, etc.; 9 = Support to spouse/partner; 10 = Support to children; 11 = Support to grandchildren; 12 = Support to other relatives; 13 = Support to friends/acquaintances.

### 3.5 Factor analysis of the associations between receiving and providing support: post-correlation examination

To further explore the associations between support forms, we have employed a post-correlation factor-analysis. This analysis helped us to identify latent (unobserved) variables that further explain patterns of correlations across multiple observed variables. To determine the suitability of the data for the factor analysis we looked at the Kaiser-Meyer-Olkin (KMO = 0.737) measure of sampling adequacy and Bartlett’s test of sphericity (χ^2^ = 761.846, df = 78, *p* < 0.001). Furthermore, to assess how well the extracted factor structure fits the data, we performed a goodness of fit test. The chi-square goodness-of-fit test showed that the model fit the data moderately well, χ^2^(42) = 77.923, *p* = 0.001. Using maximum likelihood factor analysis, we extracted the number of factors relevant for the analysis using eigenvalues greater than one and the cumulative variance that the extracted factors explain. In our case, 3 distinct factors explained 56.532% of the variance (unrotated), however, they explained 45.652% of the variance in the rotated version.^3^

The pattern matrix (Table 7) revealed three latent dimensions of social support. Factor one showed particularly high loadings with items related to support from formal structures such as social services professionals, counseling/contact/information center, family caregiver groups *(peer support through support group, online forums, etc*.*)* and health/medical professionals; hence, we have labeled factor one as “receiving formal and institutional support.” Factor two showed high loadings with forms of providing support such as friends, neighbors, and fellow club/association members, therefore, we have labeled factor two as “Providing community and social network support”. Last, factor three showed high loadings with providing support to children and grandchildren, therefore, we have labeled factor three as “providing family-support.”

**Table 7:** Pattern matrix.

Variable	Receiving formal and institutional support	Providing community and social network support	Providing family support
Social services professionals (RS)	0.845		
Counseling/contact/information center *(i.e., Pro Infirmis, municipality)* (RS)	0.844		
Family caregiver groups *(peer support through support group, online forums, etc.)* (RS)	0.716		
Medical/health professionals (doctor, nurse, psychologist, etc.) (RS)	0.658		
Friends/acquaintances (PS)		0.771	
Neighbors (PS)		0.714	
Fellow club/association members (PS)		0.525	
Volunteers (neighborhood assistance, religious community, etc.) (RS)	0.425	0.426	
People from family/acquaintances (friends, neighbors, etc.) (RS)		0.361	
Other relatives (PS)		0.338	
Children (PS)			0.870
Grandchildren (PS)			0.729
Spouse/partner (PS)			

Extraction method: Maximum Likelihood. Rotation method: Promax with Kaiser normalization. The rotation converged in 4 iterations. Reporting rule. We report only loadings ≥ 0.30 (absolute value) and suppress smaller coefficients to keep the solution readable and focus on salient item–factor relations. PS is short for providing support, RS is short for receiving support.

### 3.6 Correlations between support receiving and providing with self-rated health and general self-efficacy

General self-efficacy showed positive correlations with 2 out of 13 forms of support receiving and giving: providing support to friends and to fellow club/association members. On the other hand, self-rated health demonstrated negative correlations with 5 out of 13 forms of support. The most notable of these was the negative correlation with support received from medical/health professionals. Additional negative correlations were observed with receiving support from family caregiver groups (peer support through support group, online forums, etc.), social services professionals, people from family/acquaintances, and volunteers. More details, including sample sizes (*n*), are presented in Table 8.

**Table 8:** Significant correlations between general self-efficacy, self-rated health, and various forms of receiving and providing support.

Variable 1	Variable 2	*r*	*P*	*N*
General self-efficacy	Fellow club/association members(PS)	0.149*	0.023	231
General self-efficacy	Friends/acquaintances (PS)	0.150*	0.016	255
Self-rated health	Family caregiving groups *(peer support through support group, online forums, etc.)* (RS)	−0.186**	0.004	238
Self-rated health	Medical/health professionals (RS)	−0.304**	<0.001	243
Self-rated health	Social services professionals (RS)	−0.169**	0.010	231
Self-rated health	People from family/acquaintances (friends, neighbors, etc.) (RS)	−0.188**	0.003	250
Self-rated health	Volunteers (neighborhood assistance, religious community, etc.) (RS)	−0.167*	0.011	234

**The correlation is significant at the 0.01 level (two-sided). *The correlation is significant at the 0.05 level (two-sided).

PS is short for providing support, RS is short for receiving support.

### 3.7 Kruskal-Wallis and Jonckheere-Terpstra tests for associations between self-rated health, self-efficacy, and support

To further explore the correlations detected above (see Table 8) between self-rated health and support and between general self-efficacy and support, we conducted two further non-parametric tests, the Kruskal-Wallis and the Jonckheere-Terpstra tests. While Spearman correlations allowed us to see overall strength and direction of associations between the variables, the Kruskal-Wallis and Jonckheere-Terpstra tests allowed us to examine specific differences between sub-groups, particularly in terms of group distributions and ordered trends.

The Kruskal-Wallis test showed interesting trends in the differences between self-rated health categories across various sources of received support. For support received from family/acquaintances (friends, neighbors, etc.), the differences across self-rated health groups were significant, χ^2^(4) = 11.99, *p* = 0.017. Participants with excellent self-rated health reported the lowest mean ranks of perceived family support, whereas those in not so good self-rated health the highest, even higher than those who reported bad self-rated health. Support received from volunteers also showed significant differences across health groups, χ^2^(4) = 10.94, *p* = 0.027. Participants with not so good self-reported health reported the highest rank mean for volunteers support, followed by those reporting good, very good and excellent health, while those with bad self-rated health reported the lowest mean ranks. In terms of support from medical/health professionals, differences were especially pronounced, χ^2^(4) = 26.39, *p* < 0.001. Participants with bad self-rated health reported highest mean ranks, followed by those with good, very good and excellent self-rated health.

Those with very good and excellent self-rated health reported substantially lower mean ranks. Support from social services professionals also varied significantly by self-rated health category, χ^2^(4) = 10.06, *p* = 0.039. The highest mean rank was reported by those with bad self-rated health, followed by participants in good, not so good, very good and excellent health, who reported the lowest mean ranks. Lastly, for support from family caregiving groups *(peer support through support group, online forums, etc.)*, the result approached statistical significance, χ^2^(4) = 9.36, *p* = 0.053. Mean rank was highest among participants with bad self-rated health, and gradually decreased across the categories, with the lowest mean ranks reported by those in very good and excellent health. All details are presented in Table 9.

**Table 9:** Kruskal-Wallis test results for receiving support and self-rated Health.

Support source	Health category	*N*	Mean rank	χ^2^(4)	*p*
People from family/acquaintances (friends, neighbors, etc.) (RS)	Bad	3	133.67		
	Not so good	27	158.89		
	Good	109	127.23		
	Very good	87	121.02		
	Excellent	24	95.27	11.99	0.017
Volunteers (neighborhood assistance, religious community, etc.) (RS)	Bad	3	84.00		
	Not so good	26	143.77		
	Good	100	119.91		
	Very good	82	112.50		
	Excellent	23	99.52	10.94	0.027
Medical/health professionals (RS)	Bad	4	152.38		
	Not so good	26	141.52		
	Good	106	135.38		
	Very good	84	111.82		
	Excellent	23	70.15	26.39	<0.001
Social services professionals (RS)	Bad	4	134.13		
	Not so good	25	115.34		
	Good	97	122.68		
	Very good	83	110.17		
	Excellent	22	106.00	10.06	0.039
Family caregiving groups *(peer support through support group, online forums, etc.).* (RS)	Bad	3	149.83		
	Not so good	26	129.02		
	Good	103	123.89		
	Very good	83	112.04		
	Excellent	23	112.04	9.36	0.053

The Kruskal-Wallis’s test was used because it is appropriate for comparing differences between multiple groups (self-rated health categories) when the data is non-normally distributed.

Through the Jonckheere-Terpstra test we examined ordered trends in general self-efficacy scores based on the frequency of providing support to friends/acquaintances and fellow club/association members, which were the only significant correlations observed between the respective variables. For support to friends/acquaintances, the test showed a statistically significant positive ordered trend (Z = 2.33, *p* = 0.020), meaning that self-efficacy scores increased across higher levels of support provision. A similar significant positive ordered trend was observed for providing support to fellow club/association members (Z = 2.29, *p* = 0.022), again showing general self-efficacy increasing across higher levels of support provision. Detailed results are presented in Table 10.

**Table 10:** Jonckheere-Terpstra test for trends in self-efficacy scores by support providing frequency.

Variable	Providing support to friends/acquaintances	Providing support to fellow club/association members
Number of levels in self efficacy	22	21
Observed J-T statistic	16,274.500	13,463.500
Mean of J-T statistic	14,921.500	12,239.000
Standard deviation of J-T statistic	580.715	534.520
Standardized J-T statistic	2.330	2.291
Asymptotic significance (2-sided)	0.020	0.022

The Jonckheere-Terpstra test was used to assess ordered trends in general self-efficacy scores across the frequency of support provision, as it is designed for ordinal data with a natural order.

## 4 Discussion

First, we explored older adults’ patterns in three domains: receiving and providing support across various sources, self-rated health, and general self-efficacy. Participants in our study reported high levels of general self-efficacy (M = 31.92), which is slightly higher than the international mean (29.55) observed in a large cross-cultural sample of 19,000 individuals from 25 countries (Scholz et al., 2002), measured through the same scale applied in our study; the General Self-Efficacy Scale by Schwarzer and Jerusalem (1995). Similarly, around 89% of participants in our study reported their self-rated health as good, very good, or excellent. These results further confirm and also excel national data that shows that older adults in Switzerland perceive themselves as healthy. According to a 2025(a) report by the Swiss Federal Statistical Office, 79% of Swiss residing adults aged 65–74 years reported very good health, while this figure declined to 69% among those 75 years and older. One noticeable reason why the self-rated health may be higher in our study, compared to the Swiss national report mentioned above, may also be because the sample in our study might likely be more homogenous in relation to socio-demographics, with moderate to high educational attainment and stable financial conditions (Table 2). Our participants also reported that they provided more support than they received. Nearly half of them reported that they support their spouses/partners weekly or daily, and they also provided support to children, grandchildren, friends/acquaintances, and neighbors. We have also observed clear differences between the three systems: Informal personal ties, civic & peer organizations, and professional/institutional services (trained or mandated providers). The informal personal ties system, particularly through the support provided to in-kin partners, children, and grandchildren, was the most prominent, followed by other individuals such as neighbors and friends. The most distant were the more formal actors, such as counseling/contact/information center and medical/health professionals. The logic behind these trends may be explained by the fact that the informal personal ties environment is the most immediate both physically and emotionally, based on frequent daily interactions, such as those with a partner. Interactions with the civic-peer organizations are more peripheral, while interactions with formal support sources are least frequent, in this case, in terms of receiving support from them. In our sample, this may not necessarily indicate a lack of access but rather that participants may simply feel they do not require formal help at this stage, supported by their levels of general self-efficacy and self-rated health. This distinction from close to more distant relationships can also be also understood from the socioemotional selectivity theory, according to which, particularly in older age, because of their perceptions of their sense that they have less time ahead in life, individuals choose to prioritize relationships that are more meaningful to them, mostly emotionally (Carstensen et al., 1999). Hence, it is to be expected that personal-informal ties context is the one with which they interact the most even in the support exchanges. When we compare the results on support receiving and giving and most noticeable aspects, such as the contributions older adults make to their families, we corroborate previous national reports and documents. While 53 participants in our study reported providing support for grandchildren once a month (53/231 = 22.9%), national reports show comparable figures of 20%–21% (Swiss Federal Statistical Office, 2023). Furthermore, although interactions with the community are less frequent compared to those within the home, a considerable number of participants still supported others in the community. Specifically, 45 out of 263 participants (valid *N*) reported providing support to their neighbors, and 60 out of 266 participants (valid *N*) reported providing support to their friends/acquaintances, once a month. However, it is important to take into account that solidarity-based community engagement is only one of the ways older adults interact within their communities, other forms include nature pursuits and participation in organizations, as shown in our study published earlier this year (Gashi et al., 2025).

We also looked at how specific forms of support, across the three systems correlate with one another. The first observation is that personal-informal ties and civic & peer organizations sources interact in between and across. When focusing on interactions between informal personal ties and civic-peer organizations, we have observed, for example, that providing support to friends correlated highly with supporting neighbors and moderately with supporting fellow club/association members which shows a tendency of community support forms to cluster across its forms. The co-existence of various interaction forms that characterize the community-based exchanges of older adults is also shown in studies that look at social participation. For example, Hank and Stuck (2008) showed that those who volunteer are also likely to support others in their communities, or other researchers found positive associations between attending cultural events and engaging in gardening with maintaining regular phone contact with others (Yılmaz et al., 2025), or between involvement in political activities with the group-oriented and productive ones (Bukov et al., 2002).

Particularly, intergenerational support is shown to be an “island” of its own in our study, when support provided to children correlates high with support provided to grandchildren but correlates low (below 0.3) with other forms of support, including others in the informal personal ties system, such as providing support to the partner. Support to grandchildren is also help to one’s children and can be seen as an act that benefits both generations at the same time. This dual function is also reflected in an earlier study by Huo et al. (2018), who found that grandparents’ support for grandchildren depend not only on their own ties but also on the parents’ needs, irrespective of other factors, which can potentially also explain the low correlations to other forms of support. Our results also show small associations between support based on informal and personal ties with professional/institutional services, such as for example receiving support from family and health /medical professionals, which correlated moderately, however other associations between the two systems are either non-significant, or significant but small in power.

The last part of the results described above also reflects conclusions previous research about the “unclear” and “multilayered” relationships between informal caregivers and formal care actors and professionals. Informal caregivers are recognized by professionals as relevant resources (Dal Bello-Haas et al., 2014); however, actual collaboration is rare, because of hierarchical perceptions among professionals (McWilliam et al., 2001), and what happens is that informal caregivers end up doing the “work,” of a professional (Lyu et al., 2024).

Interestingly, one particular form of support, that of receiving support from volunteers seems to be interacted both with the system of informal-personal ties correlating with receiving support from family, but also associates moderately with various formal systems, such as support from family caregiving groups *(peer support through support group, online forums, etc.)*, health/medical experts, and social services professionals. Hence, there is an argument to be made but also be further explored in future research that integrating volunteers in the process of care can potentially influence the interactions between home and formal care actors. This has been documented in a study by Gaber et al. (2020), which included volunteers, most of them retired health professionals, in the primary care team, assisting them with information collection, communication with the clients, etc. The authors of this study concluded that this had a positive impact on the care process, as volunteers became a helpful part of the team.

The factor analysis we conducted, further confirmed patterns observed above in the correlation analysis. First, it reinforced the internal coherence of the support provided in the community, where strong correlations between support to friends, neighbors, and fellow club/association members are reflected in their high loadings on a single latent factor (factor 2). Second, the factor analysis also supports the idea that intergenerational family support is isolated as support to children and grandchildren clustered highly in a separate factor (3), just as the correlational analysis also indicated. Third, the distinction of formal and institutional support is supported (factor 1), as items related to professional and institutional actors loaded together on a separate factor, aligning with their limited correlations particularly with informal personal ties support. Last, the cross-loading of the volunteer support item across both community (factor 2) and formal (factor 1), highlights the “bridging” role suggested by the correlational analysis.

Lastly, in our study we aimed to examine the interactions between determinants of functional ability, environment (support given and received) with intrinsic psychological domain (general self-efficacy and self-rated health) within the framework of functional ability (WHO, 2020).

Regarding the associations with general self-efficacy, our results show a selective pattern of associations rather than a broad, uniform relationship across support contexts. Out of 13 forms of support, only giving support to friends and to fellow club/association members showed a significant positive association with general self-efficacy, which we interpret as a substantive empirical finding: the association is not absent, but concentrated in specific forms of active, voluntary support provision. This pattern suggests that the key contribution lies in the selectivity of associations, rather than in a general lack of associations between general self-efficacy and support. To further research the two significant associations, we conducted the Jonckheere-Terpstra test, which confirmed a significant ordered trend in which general self-efficacy increases across ordered groups of increasing support frequency. In general, our results here show and suggest that general self-efficacy is not broadly associated across different social environments, and is far from universal, depending on the type of support and its accompanying factors. We interpret this as an empirical insight on general self-efficacy, suggesting that even a “general” sense of efficacy in older age is shaped by social contexts and is more likely to be expressed in active, voluntary roles, such as supporting friends or being active in associations, rather than across all support contexts. This lines up with the meta-analysis by Whitehall et al. (2021) on general self-efficacy in older adults receiving care in different settings. The results of this study showed that depending on the form of care older adults receive, their general self-efficacy varies, with those in acute care having the lowest general self-efficacy scores, while those who received care from primary care providers reported the highest. The takeaway here is that general self-efficacy is not automatically high just because medical support is involved. It depends on context. The same with our results: out of 13 forms of receiving and providing support, only two forms of providing support correlated positively. Our results also corroborated and added to previous findings from Hosseingholizadeh et al. (2019). Hosseingholizadeh et al. (2019) show how GSE, and perceived support jointly promote social participation, while our results specify that the act of giving support within the surrounding social contexts to close ties like friends and structured groups like associations, are associated positively with general self-efficacy.

Regarding the associations between social support and self-rated health, our results showed a partial confirmation of the hypothesis, because it correlated negatively with 5 out of 6 forms of receiving support, with the exception of support from counseling/contact/information center but showed no significant correlations with forms of providing support. The positive correlations were both with official and formal sources of support such as support from medical health carers, social work health carers and family caregiving groups *(peer support through support group, online forums, etc.)*, and with less formal support such as support from family and volunteers.

Referring to previous research, our results are supported by other studies finding positive associations between self-rated health and received support (i.e., White et al., 2009; Bélanger et al., 2016; Zunzunegui et al., 2001; Mao et al., 2020). The additional analysis of Kruskal-Wallis revealed a more complex view of those associations’ variations across health categories and forms of support.

Rather than a linear increase in support as the self-rated health worsens, the distribution differs by source. Support received from medical/health professionals and family caregiving groups (peer support through support group, online forums, etc.) shows a monotonic pattern in mean ranks, with the highest mean ranks at bad self-rated health and progressively lower mean ranks in better self-rated health categories. However, social services professionals, family/acquaintances (friends, neighbors, etc.) (RS) and volunteer support, showed a non-monotonic patter, with mean ranks rising toward not so good self-rated health and dropping in the bad category. Support from social service professionals also varied across health categories and did not follow a uniformly monotonic pattern, with ranks also shifting across good self-rated health.

We argue that these mean rank patterns, are consistent with a need-graded logic (higher mean ranks among worse self-rated health), while others are consistent with a threshold/visibility logic, where mean ranks rise and peak around not so good self-rated health, when needs may become more visible, and then drop again in the bad health category. The presence of multiple logics of support can also be described with the principles of the convoy model (Antonucci et al., 2010), when different parts of the social network respond differently depending on need, timing, and closeness.

## 5 Conclusion

Despite its limitations, such as using an online-only, German-language survey, and a relatively homogenous sample, our findings generate useful inputs for future research. Methodically, we find that it is important to mention that we chose not to apply corrections as a follow-up analysis to multiple tested correlations to reduce the risk of Type II errors (false negatives). This means that there was a tradeoff to be made, and we have done it at the expense of Type 1 errors (false positives), because we think that given the exploratory nature of the study, this tradeoff was necessary to avoid missing potentially important initial associations, which could be tested and replicated in later studies. Despite these limitations, our findings provide valuable insights for future research. We believe that this is the one area in which our research contributes most, i.e., by providing directions on how and where additional knowledge is needed, regarding associations between support received and given, general self-efficacy (or specific self-efficacy), and self-rated health. Hence, we have generated some research questions that we think have the potential to further advance research in the field. For example, why are there support provided to friends and to fellow club/association members related to general self-efficacy, while others, including those support interactions with family or neighbors, are not? Do these two forms provide specific benefits, such as a sense of accomplishment, more than others, or what unites these two forms of providing forms to correlate positively with general self-efficacy? What differentiates them from the other forms of support? Or are the positive correlations detected in our study replicable or a Type I error consequence? This result also challenges the potential of general self-efficacy measures to capture the associations with support systems and can be further tested by using support-specific self-efficacy and checking if it captures associations differently. The negative associations between receiving support and self-rated health can also be further explored in future research, preferably utilizing qualitative methods. Do these associations mainly reflect changes in self-rated health itself, or do they also reflect how receiving support is interpreted and experienced, for example as a signal of dependency, vulnerability, or internalized stigma. Building on the Kruskal-Wallis mean-rank patterns, a further question is why some sources of support show the highest mean ranks in the bad self-rated health category, while others peak at not so good self-rated health and then drop at bad. Does this reflect differences in how support is mobilized as self-rated health worsens, such as shifts from informal to formal sources, changing visibility of need, or reduced access to certain types of support? Last, our findings indirectly pose questions on the social meaning of help-receiving and its interactions with personal identity and dignity and society expectations.

## Data Availability

The raw data supporting the conclusions of this article will be made available by the authors, without undue reservation.

## Footnotes

1 Our second and third studies were conducted using the same participant cohort and procedural infrastructure.

2
https://elearning.hslu.ch/ilias/login.php

3 Unrotated factors maximize variance sequentially, while rotation reorients the axes to simplify the loading pattern by redistributing variance across factors, which is why the rotated cumulative percentage can be lower (especially with oblique Promax rotation).
